# Metal Additive Manufacturing: Design, Performance, and Applications

**DOI:** 10.3390/ma18030545

**Published:** 2025-01-25

**Authors:** Nikolaos Kladovasilakis, Konstantinos Tsongas, Dimitrios Tzetzis

**Affiliations:** 1 Centre for Research and Technology Hellas, Information Technologies Institute (CERTH/ITI), 57001 Thessaloniki, Greece; 2 Advanced Materials and Manufacturing Technologies Laboratory, Department of Industrial Engineering and Management, School of Engineering, International Hellenic University, 57001 Thessaloniki, Greece; 3 Digital Manufacturing and Materials Characterization Laboratory, School of Science and Technology, International Hellenic University, 14th Km Thessaloniki-Moudania, Thermi, 57001 Thessaloniki, Greece

The rapid evolution of metal additive manufacturing (AM) has led to transformative advancements across various industries, including aerospace, healthcare, automotive, and energy. While this groundbreaking technology holds immense promise, its widespread adoption faces significant challenges that demand innovative solutions in design, performance optimization, and application-specific development. The Special Issue titled “Metal Additive Manufacturing: Design, Performance, and Applications” addresses these challenges by serving as a dedicated platform for disseminating cutting-edge research and pioneering insights. By presenting novel methodologies, rigorously evaluating material properties, and showcasing state-of-the-art real-life applications, this collection of high-quality scientific papers aims to deepen our understanding and accelerate the implementation of metal AM in industry.

This Special Issue comprises 10 high-quality papers authored by 47 contributors from 22 distinguished institutes and organizations worldwide, reflecting the collaborative and interdisciplinary nature of this Special Issue. The studies featured in this Special Issue build on a foundation of 381 conducted and published works, culminating in comprehensive and novel findings that advance the field. The significant response from the scientific community further underscores the significance of this Special Issue, with over 9600 reads recorded within the first nine months of its release.

The published papers in this Special Issue were balanced between metal AM product design, performance, and applications. In detail, Liu H. et al. [1] contributed a paper entitled “Thermal behavior and mechanical properties of different lattice structures fabricated by selective laser melting”, providing research findings on the design and performance of metal AM. Xia P. et al. [2] contributed a paper entitled “Study on Microstructure and Thermal Cracking Sensitivity of Deposited Ti6Al4V/Inconel 718 Composites Made by Two-Wire Arc Additive Manufacturing by Current”, providing research findings on the performance of metal AM. Lee J. et al. [3] contributed a paper entitled “Study On Surface Quality Analysis Of An Uncoated Boron Steel And Its Oxide Layer Suppression Method For Hot Stamping”, providing research findings on the performance of metal AM. Perrone A. et al. [4] contributed a paper entitled “Various Configurations for Improving the Efficiency of Metallic and Superconducting Photocathodes Prepared by Pulsed Laser Deposition: A Comparative Review”, providing research findings on the performance and applications of metal AM. Giarmas E. et al. [5] contributed a paper entitled “Nanoindentation Creep Behavior of Additively Manufactured H13 Steel by Utilizing Selective Laser Melting Technology”, providing research findings on performance and applications of metal AM. Egorov S. et al. [6] contributed a paper entitled “Effect of the Cooling Liquid on the Milled Interface in the Combined Process of Milling and Direct Metal Deposition”, providing research findings on performance and applications of metal AM. Kim S. et al. [7] contributed a paper entitled “Accuracy of Mandibular Removable Partial Denture Frameworks Fabricated by 3D Printing and Conventional Techniques”, providing research findings on the performance and applications of metal AM. Kladovasilakis N. et al. [8] contributed a paper entitled “Metal 3D-Printed Bioinspired Lattice Elevator Braking Pads for Enhanced Dynamic Friction Performance”, providing research findings in the design and applications of metal AM. Zykona A. et al. [9] contributed a paper entitled “Effect of “ColdArc” WAAM Regime and Arc Torch Weaving on Microstructure and Properties of As-Built and Subtransus Quenched Ti-6Al-4V”, providing research findings in applications of metal AM. Finally, Hawryluk M. et al. [10] contributed a paper entitled “Improvement of the technology of precision forging of connecting rod-type forgings in multiple systems, in the aspect of the possibilities of process robotization by means of numerical modeling”, providing research findings on the performance and applications of metal AM.

This Special Issue not only advances the scientific understanding of metal AM but also serves as a bridge between research innovation and industrial application. We extend our sincere gratitude to all the authors who contributed their exceptional work to this Special Issue. Their valuable research and dedication have been instrumental in shaping the success of this collection. We also thank the reviewers for their invaluable expertise in ensuring the quality and rigor of the published studies. Finally, we thank the editorial team for their unwavering support and commitment throughout this process. Together, these efforts have created a meaningful contribution to the field and will undoubtedly inspire further advancements in metal AM.
